# Is Accurate Dental Implant Placement Feasible Using a Novel Dynamic Computer‐Assisted Surgery System Without Patient Optical Markers or Registration? A Preliminary Retrospective Cohort Study

**DOI:** 10.1002/cre2.70256

**Published:** 2026-02-17

**Authors:** Gang He, Hongbing Liao, Hong Sheng, Eduard Valmaseda‐Castellón, Rui Figueiredo

**Affiliations:** ^1^ Sunshine Dental Clinic Ningbo China; ^2^ Professor of the Department of Prosthodontics, College and Hospital of Stomatology Guangxi Medical University Nanning China; ^3^ Chief Engineer of the Department of Research and Development Suzhou Digital‐Health Care Co. Ltd. Suzhou China; ^4^ Full professor of Oral Surgery and Implantology, Director of the Department of Dentistry, Faculty of Medicine and Health Sciences, Universitat de Barcelona Researcher of the IDIBELL Research Institute Barcelona Spain; ^5^ Associate professor of Oral Surgery and Implantology, Faculty of Medicine and Health Sciences, Universitat de Barcelona Researcher of the IDIBELL Research Institute Barcelona Spain

**Keywords:** accuracy, computer‐assisted surgery, dental implants, surgical navigation systems, treatment outcomes

## Abstract

**Objectives:**

To evaluate the accuracy and surgery time of dental implant placement using a novel dynamic computer‐assisted implant surgery (dCAIS) system that eliminates the need for patient registration and optical tracking markers. The secondary objective was to compare these outcomes with those obtained using a conventional dCAIS system.

**Materials and Methods:**

A preliminary retrospective cohort study was conducted involving 33 participants (33 implants). Eleven implants were placed using the novel dCAIS system that determines patient positioning based on anterior tooth anatomy (Prototype group), while 22 implants were placed using a conventional dCAIS system requiring standard registration and an optical tracking marker (Control group). Pre‐ and postoperative cone‐beam computed tomography (CBCT) scans were superimposed to assess implant placement accuracy. Descriptive and bivariate analyses were performed to compare accuracy and surgery time between the two groups.

**Results:**

Mean angular deviations were similar between groups (*p* = 0.924): 1.95° (SD 1.38) in the prototype group and 2.38° (SD 2.30) in the control group. No significant differences were observed in platform global deviation (mean difference [MD]: −0.33 mm; 95% CI: −0.75 to 0.09), apex global deviation (MD: −0.43 mm; 95% CI: −0.94 to 0.08), or apex depth deviation (MD: 0.28 mm; 95% CI: −0.30 to 0.86). Surgical procedures were significantly faster in the prototype group (*p* = 0.002; MD: 3.0 min; 95% CI: 0.56–5.45).

**Conclusions:**

The findings of this preliminary study seem to suggest that the tested prototype dCAIS system may be feasible to accurately place implants without conventional registration or optical tracking, potentially reducing surgical time. However, these findings should be interpreted with caution due to the study limitations.

## 1 Introduction

A prosthetically driven approach to dental implant placement is essential to meet the esthetic and functional demands of the patient (Jorba‐Garcia et al. 2025). Implant deviations may lead to intraoperative injuries to critical anatomical structures such as the inferior alveolar nerve (Vázquez‐Delgado et al. 2018) and have also been associated with a higher risk of long‐term biological complications, including peri‐implantitis (Monje et al. 2019).

The use of computer‐assisted implant surgery (CAIS) systems has increased in recent years. While static CAIS (sCAIS) methods rely on surgical guides to accurately position implants, dynamic CAIS (dCAIS) systems—also known as navigation systems—provide real‐time feedback on the position of drills and implants relative to the virtual preoperative plan (Xu et al. 2024; Khaohoen et al. 2025; Jorba‐García et al. 2023).

Several meta‐analyses have demonstrated that dCAIS systems are reliable and enable accurate implant placement without clinically significant deviations (Khaohoen et al. 2025; Yu et al. 2023; Jorba‐García et al. 2022; Mahardawi et al. 2024). According to a randomized clinical trial conducted by Jorba‐García et al. (2023), the use of dCAIS significantly reduced mean angular (mean difference [MD] = 3.9°), global platform (MD = 1.1 mm), and global apex (MD = 1.4 mm) deviations compared to the traditional non‐guided freehand approach.

However, navigation systems also have limitations. They require costly equipment and specific training (Xu et al. 2023; Block et al. 2017), and surgical procedures are often prolonged due to the need for registration and calibration steps (Jorba‐García et al. 2023).

Moreover, all commercially available dCAIS systems require the attachment of optical trackers to both the handpiece and the patient. These trackers are essential for identifying the position of the patient and instruments relative to the cone‐beam computed tomography (CBCT) images and the preoperative plan (Eggers et al. 2006). However, the patient‐mounted tracker may obstruct the surgeon's view, reduce patient comfort, and, if accidentally displaced, lead to clinically relevant inaccuracies.

A dCAIS system that does not require a patient tracker could therefore offer significant clinical advantages. Thus, the aim of the present study was to evaluate the accuracy and surgery time of dental implant placement using a newly developed dCAIS prototype that registers the patient's position based on the anterior tooth anatomy. These outcomes were compared with those obtained using a conventional dCAIS system that requires patient registration and the placement of an optical tracker.

## 2 Materials and Methods

A preliminary retrospective cohort study with matched controls was conducted involving patients requiring single‐unit dental implant placement who attended a private dental clinic (Sunshine Dental Clinic, Ningbo, China) between November and December 2024. All procedures were performed by a single surgeon (G.H.) with over 7 years of experience using dCAIS systems.

The study protocol was approved by the Ethics Committee of the College and Hospital of Stomatology, Guangxi Medical University (protocol number: 2025046), and was conducted in accordance with the principles of the Declaration of Helsinki (Halonen et al. 2020). As the study involved only anonymized retrospective data, the requirement for informed consent was waived. The Strengthening the Reporting of Observational Studies in Epidemiology (STROBE) guidelines (von Elm et al. 2008) were followed for reporting this study.

### 2.1 Study Population

All consecutive patients requiring single‐unit dental implant placement using a novel dCAIS system prototype (Dcarer Next‐Gen; Suzhou Digital‐Health Care Co. Ltd., Suzhou, China) were included in the Prototype group. This system uses anterior tooth anatomy for the registration process and for determining the patient's position. For each participant in the Prototype group, two matched controls were selected based on implant location (anterior/premolar/molar) and arch (maxilla/mandible). These control patients were treated with a conventional dCAIS system (Dcarer; Suzhou Digital‐Health Care Co. Ltd., Suzhou, China) at the same center, within a similar time frame (November to December 2024), and by the same surgeon. These patients were randomly selected from a list of operated patients using a random sequence. Patients with significant systemic diseases (American Society of Anesthesiologists [ASA] classification > 2) and without anterior teeth and those requiring bone or soft tissue grafting were excluded from the study.

### 2.2 Interventions

All included participants underwent an intraoral scan (Trios 3, 3‐Shape; Copenhagen, Denmark) and a preoperative CBCT scan (Newtom; Cefla, Italy; FSV: 90 kV, 3.00 mA, FOV: 100 × 100 mm; voxel size: 0.3 mm). The digital impressions and CBCT data were imported into the planning software (Dcarer planning software; Suzhou Digital‐Health Care Co. Ltd., Suzhou, China) to perform the virtual implant planning according to prosthetically driven principles. The dental implant dimension was selected according to the participant's clinical and anatomical characteristics.

All patients were prescribed Cefradine (Lukang, Jining, China) as a prophylactic antibiotic, and all surgeries were performed under local anesthesia using 4% articaine with 1:100,000 epinephrine (Primacaine, Acteon, Merignac, France).

#### 2.2.1 Prototype Group

**Figure 1:**
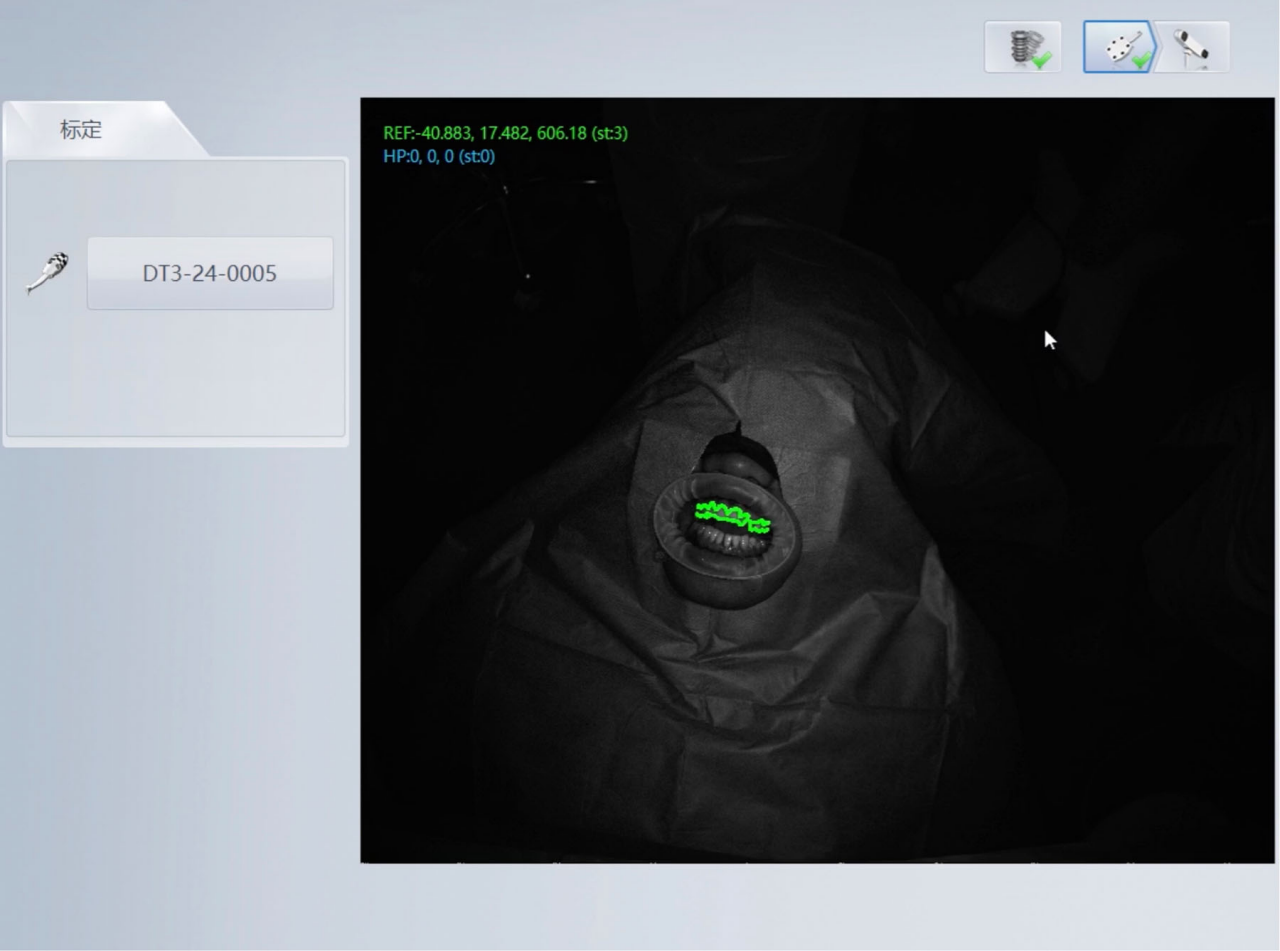
Prototype dCAIS system. This device automatically recognizes the patient's position using the anatomical features of the anterior teeth.

In the Prototype group (test group), no patient registration process or optical marker attachment was required, as the system automatically recognized the patient's position using the anatomical features of the anterior teeth (Figure 1). Additionally, bur and implant registration were not necessary, since the system includes an integrated library with data for various dental implant manufacturers. Accuracy was verified by touching specific anatomical landmarks with the surgical bur. This verification step was repeated each time the bur was changed, to ensure continued precision.

**Figure 2:**
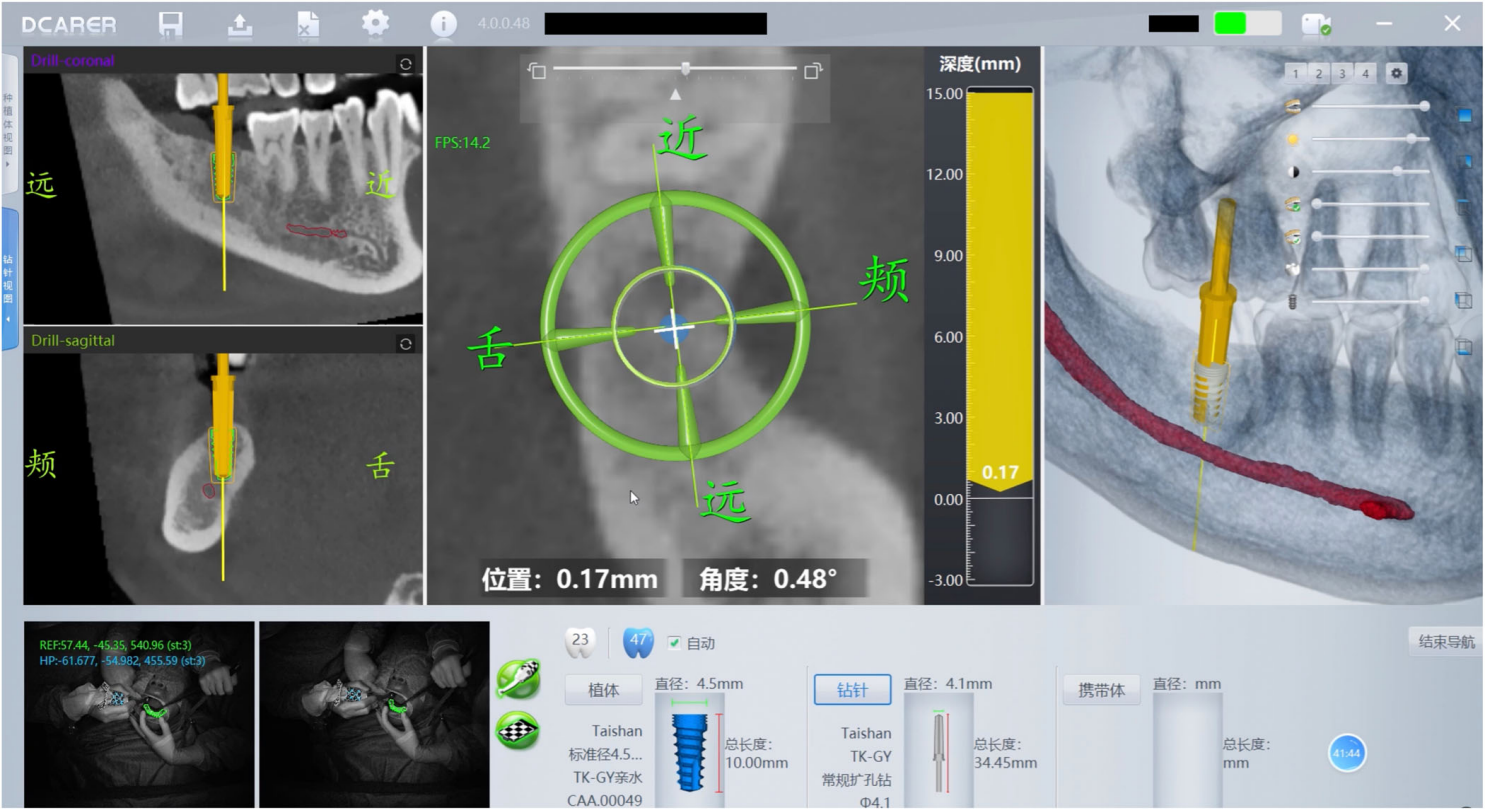
Real‐time guidance displayed on the computer screen of the prototype dCAIS system.

Subsequently, implant bed preparation and implant placement were performed under real‐time guidance displayed on the computer screen of the prototype dCAIS system (Dcarer Next‐Gen; Suzhou Digital‐Health Care Co. Ltd., Suzhou, China) (Figure 2). Whenever clinically feasible, a flapless surgical approach was employed.

Compared to existing dCAIS systems that rely on artificial feature‐based reference locators for positioning, the new prototype integrates machine vision and artificial intelligence (AI) to achieve high‐precision, real‐time, and highly robust tracking of weakly textured dental objects. The innovative algorithms are as follows: using intraoral scan data as a bridge, the prototype establishes a mapping relationship between dental navigation images and the CBCT data. First, it automatically aligns intraoral scan data with the CBCT data by combining tooth position information and contour morphology, ensuring consistency of the coordinate system. Next, an AI model analyzes the intraoral scan data to extract 3D dental features automatically. Then, a large‐scale real‐world dental image database is constructed, and a cascaded AI model is trained to extract dental features from real‐time intraoral images. By combining parameters from the optical navigation system, high‐precision mapping and pose registration between 2D dental image features and 3D intraoral scan features are achieved, enabling highly accurate dental localization.

#### 2.2.2 Control Group

In the Control group, a conventional dCAIS system (Dcarer; Suzhou Digital‐Health Care Co. Ltd., Suzhou, China) was used. During the preoperative CBCT scan, a radiographic clip (Registration device; Suzhou Digital‐Health Care Co. Ltd., Suzhou, China) was attached to the patient's teeth. Before surgery, a radiographic marker‐based registration process was carried out, and the patient tracker was positioned according to the manufacturer's instructions. Once accuracy was verified by touching a specific anatomical area with a surgical bur, a guided drilling sequence was carried out using the real‐time feedback provided by the conventional dCAIS system. As in the Prototype group, drill and implant registration were not required, as the necessary data were already stored in the dCAIS system library.

All participants in both groups underwent a postoperative CBCT scan using the same device and acquisition parameters as those used for the preoperative scan.

### 2.3 Outcome Variables

To assess deviations between the planned and actual implant positions, Digital Imaging and Communications in Medicine (DICOM) files from the pre‐ and postoperative CBCT scans were superimposed. All accuracy‐related outcome variables were automatically measured by a blinded examiner using dedicated software (Dcarer dynamic navigation accuracy verification software; Suzhou Digital‐Health Care Co. Ltd., Suzhou, China). After uploading the planning data from the preoperative CBCT and the postoperative CBCT, the software automatically identified the implant position and calculated the observed deviations. In rare cases where the software was unable to detect the implant, manual identification and alignment were meticulously performed by a blinded observer to ensure measurement integrity.

The primary outcome variable was the global apex deviation (the 3D linear distance between the apex of the planned and actual implant positions) measured in mm. The secondary outcome variables included (Jorba‐García et al. 2022):

– Global platform deviation (mm): the 3D linear distance between the center of the implant platform in the planned and actual positions.
– Angular deviation measured in degrees (°) between the long axis of the planned and final position of the implants.
– Apex depth deviation (mm): the vertical difference between the planned and actual apex positions.

Surgery time was registered using video files recorded by the navigation system software. The time elapsed from the beginning of surgery to placement of the healing abutment (or the final suture in cases where a flap was raised) was recorded for each procedure. In the Prototype group, the start time just before using the first drill was registered, while in the control group, the time needed to place the patient's optical marker was also included.

To assess intra‐examiner reliability, a random sample of 10 implants was re‐evaluated by the same examiner after a 4‐week interval. The intraclass correlation coefficient (ICC) for absolute agreement was 0.902 (95% CI: 0.632–0.975; *p* < 0.001), indicating excellent reliability.

### 2.4 Statistical Analysis

Data were analyzed using the Statistical Package for the Social Sciences, version 30.0 (SPSS; IBM Corp., Armonk, New York, the United States). A descriptive analysis was performed for all variables. The normality of scale variables was assessed using the Kolmogorov–Smirnov test. For categorical variables, group comparisons were conducted using Pearson's *χ* ^2^ tests or Fisher's exact tests when expected cell counts were insufficient. Comparisons between the two study groups regarding implant placement accuracy and surgery time were performed using the Mann–Whitney *U*‐test. The level of statistical significance was set at *p* < 0.05.

A post hoc power analysis was performed with the G*Power v.3.1.3 software (Heinrich‐ Heine Universität, Dusseldorf, Germany) to determine the achieved statistical power based on the observed effect size, sample size, and *α* level (0.05).

## 3 Results

A total of 33 implants were placed in 33 patients, with 11 implants in the Prototype group and 22 in the Control group. The main clinical characteristics of the sample are summarized in Table 1. No statistically significant differences were observed between groups regarding the baseline parameters. The post hoc analysis revealed an achieved power of 0.56 for the primary outcome variable.

**Table 1:** Main clinical features of the sample. No significant differences were found between the two study groups (*p* > 0.05) for any of the reported variables. Note that the controls were paired with the cases (Prototype group) for the variables “arch” and “position”.

		Prototype (*n* = 11)	Control (*n* = 22)	Total (*n* = 33)
Gender	Male	6 (54.5%)	13 (59.1%)	19 (57.6%)
	Female	5 (45.5%)	9 (40.9%)	14 (42.4%)
Age in years (standard deviation)		53.27 (14.42)	56.41 (14.45)	55.36 (14.29)
Implant position	Anterior	1 (9.1%)	2 (9.1%)	3 (9.1%)
	Premolar	2 (18.2%)	4 (18.2%)	6 (18.2%)
	Molar	8 (72.7%)	16 (72.7%)	24 (72.7%)
Arch	Maxilla	2 (18.2%)	4 (18.2%)	6 (18.2%)
	Mandible	9 (81.8%)	18 (81.8%)	27 (81.8%)
Smoking	No	7 (63.6%)	18 (81.8%)	25 (75.8%)
	10 cig/day or less	4 (36.4%)	4 (18.2%)	8 (24.2%)
	> 10 cig/day	0 (0%)	0 (0%)	0 (0%)
Implant brand	Nobel Biocare	1 (9.1%)	6 (27.3%)	7 (21.2%)
	Straumann	7 (63.6%)	15 (68.2%)	22 (66.7%)
	Others	3 (27.3%)	1 (4.5%)	4 (12.1%)
Implant length	< 10 mm	1 (9.1%)	6 (27.3%)	7 (21.2%)
	10–13 mm	9 (81.8%)	13 (59.1%)	22 (66.7%)
	> 13 mm	1 (9.1%)	3 (13.6%)	4 (12.1%)
Implant diameter	4.1 mm or less	4 (36.4%)	5 (22.7%)	9 (27.3%)
	> 4.1 mm	7 (63.6%)	17 (77.3%)	24 (72.7%)
Surgical approach	Flapless	11 (100%)	22 (100%)	33 (100%)
	With flap	0 (0%)	0 (0%)	0 (0%)
Site	Healed	10 (90.9%)	17 (77.3%)	27 (81.8%)
	Postextraction	1 (9.1%)	5 (22.7%)	6 (18.2%)

Across the entire sample, the median global apex deviation was 0.92 (interquartile range [IQR] = 0.59), the median angular deviation was 1.81° (IQR = 1.89), the median global platform deviation was 0.97 mm (IQR = 0.36), and the median apex depth deviation was –0.49 mm (IQR = 1.01).

**Figure 3:**
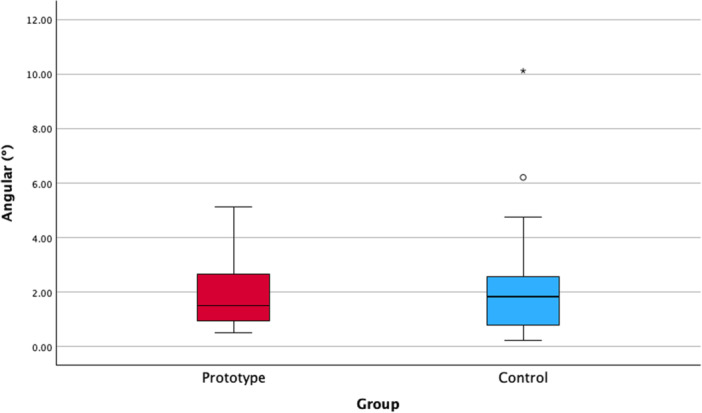
Boxplot with the angular (°) deviations registered in both groups. No statistically significant differences were found between the two study groups (Mann–Whitney *U*‐test; *p* = 0.924).

**Figure 4:**
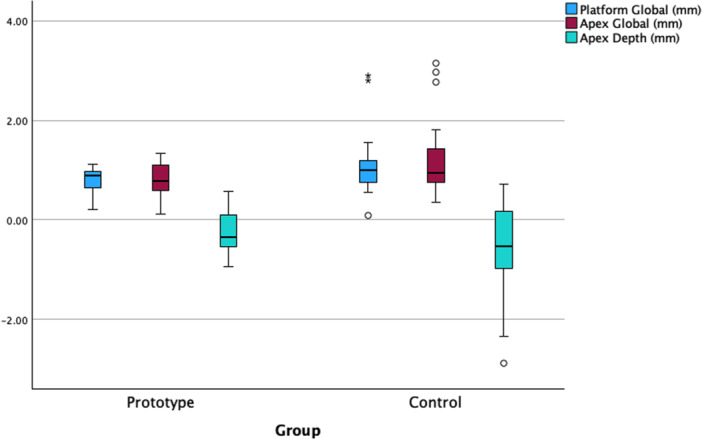
Boxplot with the linear deviations (mm) registered in both groups. No statistically significant differences were found between the two study groups (Mann–Whitney *U*‐tests; *p* > 0.05).

The accuracy outcomes for both study groups are shown in Table 2 and illustrated in Figures 3 and 4. No statistically significant differences were observed for any of the accuracy variables (*p* > 0.05). The MDs between groups were below 0.5° and 0.5 mm for all accuracy variables.

**Table 2:** Main accuracy outcomes of both study groups. No significant differences were found (Mann–Whitney *U*‐tests).

Accuracy variable	Prototype		Control		MD (95% CI)	*p* value
	Mean (SD)	Median (IQR)	Mean (SD)	Median (IQR)		
Angular (°)	1.95 (1.38)	1.50 (1.97)	2.38 (2.30)	1.82 (1.94)	−0.43 (−1.97 to 1.11)	0.924
Platform global (mm)	0.80 (0.28)	0.90 (0.50)	1.13 (0.65)	0.99 (0.51)	−0.33 (−0.75 to 0.09)	0.126
Apex global (mm)	0.80 (0.39)	0.79 (0.55)	1.23 (0.78)	0.95 (0.69)	−0.43 (−0.94 to 0.08)	0.143
Apex depth (mm)	−0.27 (0.47)	−0.35 (0.64)	−0.55 (0.87)	−0.53 (1.15)	0.28 (−0.30 to 0.86)	0.359

Abbreviations: 95% CI, 95% confidence interval; IQR, interquartile range; MD, mean difference (Prototype − Control); SD, standard deviation.

**Figure 5:**
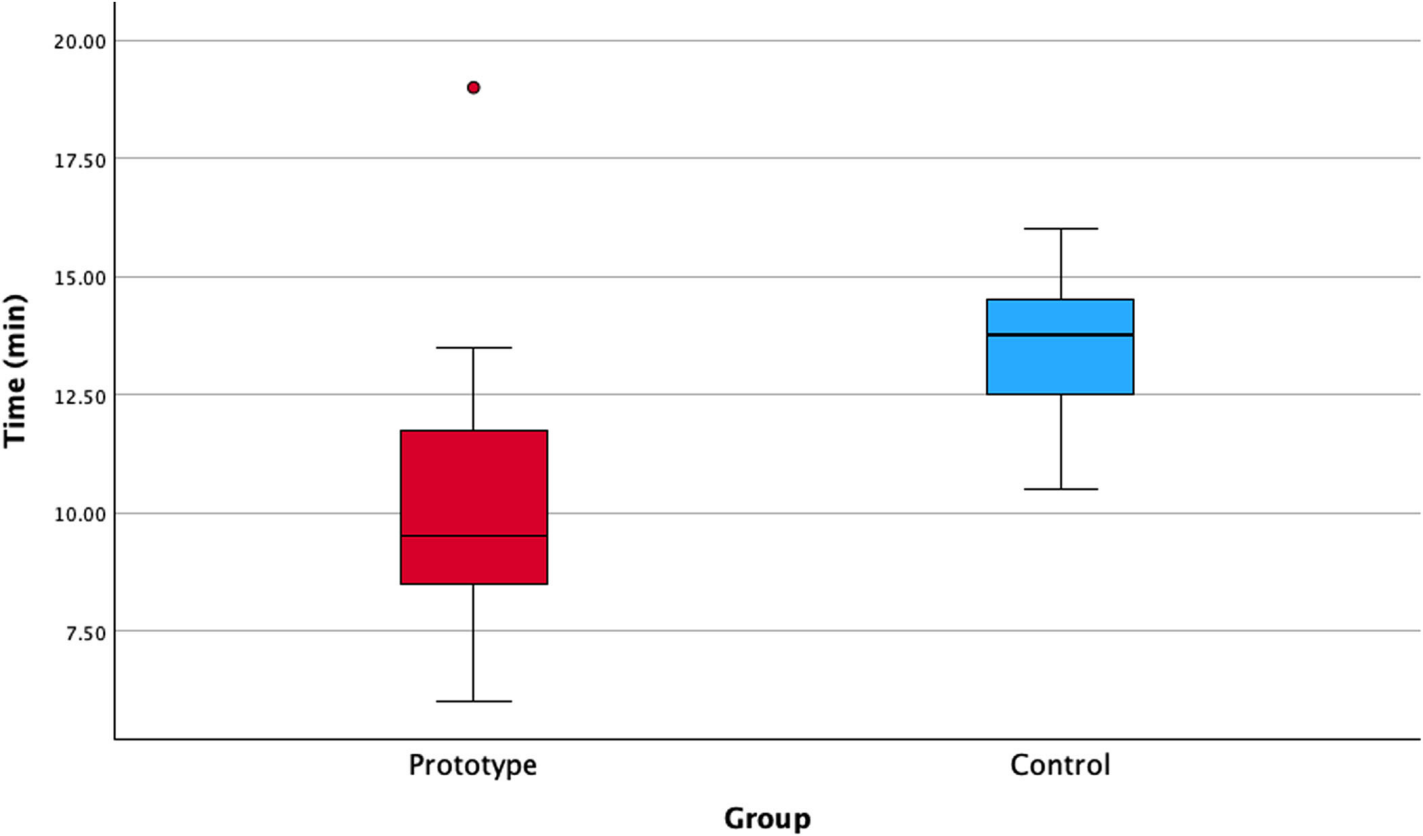
Boxplot with the surgery time (min) registered in both groups. A statistically significant difference was observed (Mann–Whitney *U*‐test; *p* = 0.002).

The median surgery time was 9.5 min (IQR = 5.0) and 13.75 min (IQR = 2.25) for the Prototype and Control groups, respectively. Thus, the surgical procedure was significantly faster in the Prototype group (MD = −3.0 min; 95% confidence interval [95% CI] = −0.56 to −5.45; *p* = 0.002) (Figure 5).

## 4 Discussion

The present study seems to indicate that accurate dental implant placement might be achievable using an innovative dCAIS system that does not require the placement of a patient‐mounted optical marker. This feature may contribute to a more comfortable experience for both patients and clinicians. Moreover, the absence of a registration step significantly reduces the duration of the surgical procedure, potentially improving clinical efficiency. Notably, the Prototype group exhibited slightly lower deviation values across all the outcome variables (Table 2).

Guided implant surgery techniques have evolved significantly over the past decades. sCAIS systems have been shown to reduce deviations during single‐tooth implant placement compared to the traditional freehand approach (Smitkarn et al. 2019). However, some studies suggest that dCAIS systems may offer even greater precision (Yu et al. 2023; Liu et al. 2024). Our results seem to confirm this statement, with a median angular deviation of 1.81° (IQR = 1.89), a median platform global deviation of 0.97 mm (IQR = 0.36), and a median apex global deviation of 0.92 mm (IQR = 0.59). Nevertheless, it remains essential to maintain a safety margin when placing implants near critical anatomical structures, as three patients in the control group exhibited apex global deviations exceeding 2 mm.

Several papers have explored potential factors that may influence the accuracy of dCAIS systems. Among the most frequently cited variables are the registration method employed (Wu et al. 2025; Jorba‐García et al. 2024a), the spatial distribution and distance between fiducial points (Choi et al. 2020; Fan et al. 2019), and the presence of radiographic artifacts (Jorba‐García et al. 2024b). The patient‐mounted optical tracker may also contribute to inaccuracies. This device must be securely attached to the patient's jaw, as the dCAIS system relies on it to determine the real‐time position of the patient during surgery. Okubo et al. (2025) reported that using a stent to stabilize the clip supporting the patient tracker significantly improved accuracy. One key advantage of the prototype evaluated in the present study is that it eliminates the need for a patient tracker. This not only removes a potential source of deviation but also enhances patient comfort and improves surgeon ergonomics. The presence of the tracker can obstruct the surgical field and prevent the patient from fully closing the mouth during the procedure. However, according to Kunavisarut et al. (2022), in participants undergoing single‐tooth replacement in posterior sites, there were no significant differences in patient‐reported outcome measures (PROMs) between conventional freehand surgery and sCAIS systems.

Surgical experience is a critical factor influencing both the complication rates and the surgery time. Caponio et al. (2025) reported a higher dental implant failure rate (5.6% vs. 3.7%) when early‐career clinicians were involved. Regarding implant placement accuracy, Jorba‐García et al. (2019) observed that dCAIS systems may reduce performance differences between novice and experienced surgeons. Nevertheless, a recent study suggested that at least three practice sessions may be beneficial to improve outcomes for both novice and experienced operators (Kundaechanont et al. 2025). In the present study, all procedures were performed by a single surgeon (G.H.) with extensive experience in implant dentistry and over 7 years of consistent use of dCAIS systems. Therefore, further research involving less experienced clinicians is warranted to assess the influence of surgical experience when using this novel dCAIS prototype.

This study presents several limitations that should be acknowledged. Firstly, the small sample size limited the statistical power of the analysis. However, the accuracy outcomes in the Prototype group were notably consistent, with slightly lower deviations compared to the Control group (Figures 3 and 4). Still, these findings should be interpreted with caution and validated in future studies with larger cohorts. Secondly, the findings are restricted to single‐tooth implants placed in the posterior region by an experienced surgeon, which limits their generalizability. Future studies involving implants in different anatomical locations are warranted to validate these results. Thirdly, some variables, like bone quality and systemic pathologies, were not assessed. Although these variables are unlikely to affect implant placement accuracy, they can influence the treatment outcome. Lastly, the retrospective design of the study could introduce potential biases. Nevertheless, it is important to emphasize that all outcome variables were recorded in an objective and reproducible manner. Specifically, pre‐ and postoperative CBCT scans were available for all cases, and accuracy measurements were obtained automatically using dedicated software.

## 5 Conclusions

The findings of this preliminary study appear to suggest that the tested prototype dCAIS system may be feasible and could enable accurate dental implant placement without the need for conventional registration or patient optical tracking. Additionally, it might contribute to reduced surgical time compared to traditional dCAIS systems, as patient registration is not required. However, given the exploratory nature of the study and its limitations, these conclusions should be interpreted with caution.

## Author Contributions

**Gang He:** conceptualization, data curation, investigation, resources, writing – review and editing, funding acquisition. **Hongbing Liao:** conceptualization, methodology, writing – review and editing, supervision. **Hong Sheng:** resources, writing – review and editing. **Eduard Valmaseda‐Castellón:** conceptualization, methodology, writing – review and editing, supervision. **Rui Figueiredo:** conceptualization, methodology, formal analysis, writing – original draft, funding acquisition.

## Ethics Statement

The study protocol was approved by the Ethics Committee of the College and Hospital of Stomatology, Guangxi Medical University (protocol number: 2025046) and was conducted in accordance with the principles of the Declaration of Helsinki.

## Consent

As the study involved only anonymized retrospective data, the requirement for informed consent was waived.

## Conflicts of Interest

The authors declare the following competing financial interests or personal relationships directly related to the manuscript: Dr. Gang He has participated in sponsored lectures by Suzhou Digital‐Health Care Co. (Suzhou, China). Dr. Hong Sheng is an employee of Suzhou Digital‐Health Care Co. Ltd. Dr. Rui Figueiredo and Dr. Eduard Valmaseda report nonfinancial support from Suzhou Digital‐Health Care Co. (Suzhou, China). They would also like to declare the following interests outside the submitted work: Dr. Gang He has participated in sponsored lectures by Nobel Biocare (Zürich, Switzerland). Dr. Hongbing Liao reports no conflict of interest. Dr. Rui Figueiredo reports grants, personal fees and nonfinancial support from MozoGrau (Valladolid, Spain), Avinent (Santpedor, Spain), Inibsa Dental (Lliçà de Vall, Spain), and Dentaid SL (Cerdanyola del Vallés, Spain); nonfinancial support and personal fees from Nobel Biocare (Zürich, Switzerland); personal fees from Geistlich Pharma AG (Wolhusen, Switzerland), BioHorizons Iberica (Madrid, Spain), Araguaney Dental (Barcelona, Spain), Septodont (Saint‐Maur‐des‐fossés, France), Dentaid SL (Cerdanyola del Vallés, Spain), Global Medical Implants (Barcelona, Spain), and Laboratorios Silanes (Mexico City, Mexico) outside the submitted work. Dr. Figueiredo has also participated as a principal investigator in a randomized clinical trial sponsored by Mundipharma (Cambridge, the United Kingdom) and in other clinical trials as a coinvestigator for Menarini Richerche (Florence, Italy), Aesculap—BBraun (Tuttlingen, Germany), and Resorba Medical gmbH (Nürnberg, Germany). Dr. Eduard Valmaseda‐Castellón reports grants, personal fees, and nonfinancial support from MozoGrau (Valladolid, Spain), Avinent (Santpedor, Spain), Inibsa Dental (Lliçà de Vall, Spain), and Dentaid SL (Cerdanyola del Vallés, Spain); nonfinancial support from Nobel Biocare (Zürich, Switzerland); and personal fees from Global Medical Implants (Barcelona, Spain) and Laboratorios Silanes (Mexico City, Mexico) outside the submitted work. Dr. Valmaseda‐Castellón has also participated as an investigator in randomized clinical trials sponsored by Geistlich Pharma AG (Wolhusen, Switzerland), Aesculap—BBraun (Tuttlingen, Germany), Mundipharma (Cambridge, the United Kingdom), Resorba Medical gmbH (Nürnberg, Germany), and Menarini Richerche (Florence, Italy).

## Data Availability

The datasets generated during and/or analyzed during the current study are available from the corresponding author upon reasonable request.
